# Veterinary anthropology: Samples from an emerging field

**DOI:** 10.3389/fvets.2023.1053256

**Published:** 2023-02-20

**Authors:** Ludek Broz, Frédéric Keck, Kerstin Weich

**Affiliations:** ^1^Czech Academy of Sciences, Institute of Ethnology, Prague, Czechia; ^2^Laboratory for Social Anthropology, Centre National de la Recherche Scientifique (CNRS), Paris, France; ^3^Unit of Ethics and Human-Animal Studies, Messerli Research Institute, University of Vienna, Medical University of Vienna, University of Veterinary Medicine, Vienna, Austria

**Keywords:** veterinarization, field work, ethnography, biosecurity, global health, care, zoonoses

## Abstract

We contribute to the growing field of veterinary humanities by promoting collaboration between veterinarians and anthropologists. Veterinary anthropology as we propose it analyzes the role of animal diseases in social life while questioning notions of animal health and human health. We distinguish three ways for veterinarians to collaborate with anthropologists, which more or less follow a chronological order. One form of collaboration requires anthropologists to bring risk perception or local knowledge on zoonoses identified by veterinarians. A more recent form of collaboration integrates veterinarians and anthropologists around the view of animals as actors in infrastructures of security. Finally, we suggest that, as veterinary expertise and its roles in contemporary societies is becoming an object of anthropological enquiry, a new space for collaboration is unfolding that enables veterinarians to see themselves through that reflexive lens of anthropological attention. Veterinary anthropology can therefore be defined as an anthropology of veterinarians and with veterinarians.

## 1. Introduction

“Whenever and wherever anything happens that involves feathers, fur, or claws, we are called to manage the situation!” Summing up his professional lifetime of experience, an Austrian vet gave this remarkable characterization of contemporary veterinary work. Feathers, fur, and claws figure as markers of the idea of “the animal.” This collective singular is frequently criticized on a conceptual level for erasing the many differences and peculiarities in the worlds of different animal species. Veterinarians experience the tension of this great diversity being lumped together into one basket in a less abstract way. Often called upon to engage any “animal problem” arising in a great variety of settings and contexts, veterinarians experience the multi-faceted worlds of a multispecies medicine, which used to be kept at a distance by use of the label “the animal.” The emerging discipline of veterinary humanities counters this status quo by exploring veterinary settings as impactful spaces of encounters between humans and animals.

The first Conference of the Network for Veterinary Humanities, held in Vienna in 2020 and resulting in this research topic, served as impressive proof of the potential for exploring the diverse meanings and conditions of “doing animal health in more-than-human-worlds.”^1^ The investigation of questions of medical ethics in veterinary contexts, for example, in the assessment of quality of life or considerations of patient autonomy and consensus-based decision-making, provokes serious challenges to the established concepts. As in medical humanities, the interplay of society and the bio-medically mediated experience of health and disease is the main topic of veterinary humanities. Therefore, veterinary humanities distinguishes itself by tracking shifts and developments in the understanding and practice of health and medicine in their intersection with human-animal relations. How are boundaries between humans and animals changed or reproduced by veterinary medicine? What is the impact of dividing veterinary students along existing hierarchies between large and small animals, pets and livestock? How are veterinary values and goals evolving, and how do they feed back into society? How are the imaginations, expectations, and affects of veterinary work translated and processed in literature and fiction?

Animals are central to veterinary settings: with their presence in and co-production of emerging health sites, they define and align medical interventions as veterinary. The (empirical) centrality of animals in veterinary settings supports the closeness between animal studies and veterinary humanities. Both share an interest in animals that is politically and ethically motivated and directed against their empirical exploitation and their academic marginalization. This motivation goes beyond an interest in the implications of animals in human medicine, such as therapeutic or infectious animals, healthy or unhealthy food made from animals, or lab animals used in the production of medical knowledge and pharmaceuticals, which are the objects of medical anthropology (1). In contrast, veterinary humanities approaches veterinary worlds by describing, for example, the emergence of multispecies health sites in public (2), the spatial order of veterinary consultations (3), or the conditions of becoming an animal patient (4), *without* denying agency and productivity to the involved non-human animals. These examples point to the yet underrepresented participation of animals in meaning-making processes related to the semiotic-materialistic dimensions of life: health, birth, disease, and death.

Investigating the ways in which medicine becomes directed to animals and shaped by them also has an impact on the location of veterinary humanities on the conceptual level of medical studies. The French medical philosopher Georges Canguilhem stated that health should be understood beyond its medical categorization as a way of instantiating norms in relation to the world. This provocation forces us to understand animal health beyond its institutionalized definition by human medicine. Veterinary humanities do not add insights about animal health to the ongoing research on human health, with a focus on biomedicine as its dominating institution. Instead, relying on animal studies and anthropological theory, it rejects confusing health with medicine, either human health with human medicine or animal health with veterinary medicine. Pushing against a re-inscription of institutional distinctions and hierarchies on the common concepts at stake—health, wellbeing, sickness, mortality—veterinary humanities asks for the ways in which these broad philosophical ideas become transformed and articulated in specific contexts. Zoonoses, therapy animals, and food hygiene are specific sites of this research, not only because humans and animals are being entangled under a medical gaze, but because they exemplify the fundamental resistance of health as a dimension of life against its categorization along species borders or institutional allocations.

We want to contribute to the growing field of veterinary humanities by supporting collaboration between veterinarians and anthropologists. Gathering contributions by anthropologists, humanities scholars, and veterinarians, this collection of articles contributes to the elaboration of an emerging common sub-field that can be called, following a conference organized in Edinburg in 2016, “veterinary anthropology”.^2^ This field studies the participation of animals in meaning-making processes about notions such as life, health, birth, disease, and death, using its own methods of investigation. Indeed, anthropologists have chosen participation as their staple method of study. Similarly, veterinary work relies on a participatory approach as a key element in the fostering and regulation of the entanglement of humans and animals around the experience of health. If anthropologists are experts in participatory observation, and veterinarians are experts in multispecies participatory medicine, they meet on the common ground where animals are entangled with humans. Veterinary anthropology is a collaboration between veterinarians and anthropologists to analyze how animals participate in social life at the incidence of diseases that question notions of animal health and human health. Tim Ingold (5) defined anthropology as “philosophy with the people in.” Similarly, we define veterinary anthropology as a philosophical reflection on human-animal relations, elaborated in collaboration with veterinarians while simultaneously observing their role in those relations. Animals are present in anthropological descriptions of various cultures/societies, due to their roles in forms of human subsistence (be it hunting or agriculture) as well as in kinship, sacrifice, witchcraft, divination, and other phenomena in which they are endowed with value and meanings far beyond basic utility. Moreover, animal diseases are not only a question of applied anthropology but also involve the theoretical core of the discipline: that is, understanding how social causality emerges out of physical causality (6). We believe that veterinarians and anthropologists may learn a lot from each other on what it means for animals to participate in human social life that is always already more-than-human.

We distinguish three ways for veterinarians to interact and collaborate with anthropologists, which more or less follow a chronological order. One form of collaboration requires anthropologists to bring risk perception or local knowledge on zoonoses identified by veterinarians. A more recent form of collaboration integrates veterinarians and anthropologists around the view of animals as actors in infrastructures of security. Finally, we suggest that, as veterinary expertise and its roles in contemporary societies is becoming an object of anthropological enquiry, a new space for collaboration unfolds that enables veterinarians to see themselves through that reflexive lens of anthropological attention. Veterinary anthropology can therefore be defined as an anthropology of veterinarians and with veterinarians (7).

## 2. Anthropology with veterinarians

### 2.1. Anthropology of risk and culture

One of the more developed areas of collaboration between veterinary science and anthropology, though within the broader frame of care for human health, is the study of zoonoses. Emerging infectious diseases are increasingly seen as a severe concern for global health, leading to constant attention from specialists in both human and veterinary epidemiology, virology, etc. Yet, while pathogens mutate randomly, crossing species barriers, zoonotic spill-overs are driven and amplified by human factors such as bushmeat hunting, industrial breeding, deforestation, or urbanization. The study of zoonoses, therefore, cannot be left to natural and health sciences alone. Rather, a thorough understanding of the above-mentioned human factors provided by social scientific approaches is needed. Furthermore, while ecological concepts of disease reservoirs are based on probabilistic models at the level of animal populations, the ethnographic method is necessary to describe qualitatively and quantitatively the interactions between humans and animals that are favorable to zoonotic transmission.

The collaboration between anthropologists and scholars of zoonoses coming from health and veterinary sciences has often been framed in the language of risk and culture. Anthropologists have been called upon to identify clues in the search for sources and vectors of pathogens such as Nipah virus (8–10). While probabilistic models of natural sciences distribute risks of emergence based on vulnerabilities (for instance, the number of bats and primates sold as bushmeat in central Africa), anthropologists study the culture in which people live and which might explain behaviors seen by epidemiologists as risky (for instance, the conviction that bats have virtues that are transmitted *via* consumption of bat meat, or the narrative in which human and non-human primates share kinship relations). Some zones of intense contact and physical proximity between humans and animals therefore could be identified as hotspots for the surveillance of zoonoses (11). The task, then, is to determine whether culture is an asset or an obstacle in risk perception, communication, and possible prevention. This approach derives from the assumption that “the perception of risk is a social process” (12), and that the selection of those natural dangers that deserve attention is culturally mediated. In a division of labor characteristic of modern sciences, the natural sciences, medicine, and veterinary medicine study “nature” while anthropology studies “culture”.

Taking avian influenza in Chattogram, Bangladesh, as a case study, Høg et al. (13) seem to be exemplary of this approach. They analyze price volatility, patron-client relationships, and behaviors of last resort as structural factors that determine the perception of risk in live poultry markets. In their view, retailers are not free agents calculating the benefits and risks of reporting sick poultry, but rather, they follow structural constraints that determine their everyday life and gestures. In fact, we can imagine going even further, asking how retailers and consumers build relations with animals under zoonotic threats. For instance, in China, buying live poultry is justified by ideas about the freshness of the chickens, which relates humans and animals to the infrastructure of the cold chain, or more often its absence (14). Hence, the practice seen as risky for zoonotic threats is fueled by the fear of those very risks. As Høg et al. argue, in order to reduce the risk, it appears necessary to change the risk environment as a whole rather than to aim at singling out risky behaviors.

The difficulty raised by this approach, in our view, is that the collaboration between veterinarians and anthropologists is one-sided: veterinarians ask questions about how humans perceive zoonotic risks, and anthropologists reply with empirical studies based on participant observation, in-depth interviews, and (less often) questionnaires. Using this approach to understand how prophylactic measures such as culling, vaccination, or isolation can be accepted by retailers and consumers is likely to offer important answers; yet it is unlikely to generate new questions in dialogue with those whose lives are studied. Moreover, the role of veterinarians in selecting the frame of risk perception is overlooked. Such research would, for instance, ask how avian influenza became a public problem in Bangladesh in contrast to Vietnam, China, or India, with different modes of relations between retailers, veterinarians, and national and provincial agriculture authorities.

### 2.2. Ethno-veterinary medicine and local knowledge

Another avenue of collaboration between veterinarians and anthropologists has been paved in ethno-veterinary medicine. This label is often used to represent two related phenomena. On the one hand, the word denotes the actual knowledge and practice of people who treat ill animals. “Ethno” in ethno-veterinary stands for local, vernacular, non-Western, and non-academic veterinary knowledge and practice. On the other hand, the term denotes the Western academic field studying these knowledges and practices. This field builds on the “combination of the time-tested field interview methods of anthropologists and linguists with the clinical skills and laboratory expertise of veterinarians” (15).

The motivation behind this branch of research seems 3 fold. First, the local knowledges and practices are perceived as possibly valuable for advancing Western bio-veterinary medicine, which is especially the case when use of medicinal plants can “aid in the finding of innovative drug sources” (16). A parallel benefit, just as in the related field of ethnobotany (17), is potential conservation of medicinal plants deemed useful by foreign researchers and local communities (18). Second, many authors note that availability of bio-veterinary medicine is in many places limited and possibly further diminishing for various reasons (16, 19). Local ethno-veterinary knowledge and practice is therefore seen as a crucial alternative that can deliver good results, and should be preserved, fostered, and nourished in and for the communities that gave rise to it, and it should find ways into the policies of “national livestock healthcare systems” (16, 20). Finally, authors recognize the “ecological approach to disease prevention” in local ethno-veterinary knowledge (15). By paying attention to environmental factors in the form of food and soil quality, as well as to the interplay of animals' behaviors with their surroundings, this approach might represent not only a cheaper but also a more sustainable and environmentally sound alternative to the use of “antibiotics or highly toxic chemical dips and other commercial pesticides with long residual effects” (15).

In the light of this last objective, McCorkle and Mathias-Mundy (15) “urge that Western veterinary medicine take a harder look not only at its ethnoscientific counterpart but also at itself.” This reflexivity seems to have its limits, though. The two authors argue that “[w]orldwide, two broad types of ethnomedical etiologies can be distinguished: natural and supernatural” (10). Hence, Western bio-medical ontology, which separates natural and supernatural as one of its founding principles, is used as a prism through which all other systems of knowledge and practice are described, while being taken for granted itself (see part four of this introduction). Anthropology has a long tradition of reflexive understanding of situations in which different presuppositions of what *is* real underlie the knowledge systems of the studier and the studied, and therefore can offer to ethno-veterinary medicine much more than fieldwork interviews and observation techniques. Conflicting ontologies beyond different causalities of animal diseases are a case in point. Elephants in zoological parks or tourism centers have recently been subjects of concern because they carried *Mycobacterium tuberculosis* in a reverse zoonosis, showing how endangered animals are dependent on their relations with humans. For mahouts working with elephants as log carriers, disease is caused by the actions of spirits (*phi)* as they move from the forest to the village, and can be observed by the size of the elephant's body rather than by sampling the trunk. The surveillance of elephants by mahouts thus involves an invisible causality (spirits regulating animal movements) and a visible causality (plants acting on the growth of the body). Mahouts do more than produce their own veterinary knowledge: they observe the plants consumed by elephants in the forest and add them to their pharmacopeia (21). Animals thus appear as co-actors in the production of knowledge.

We do not open an explicit dialogue with the field of ethno-veterinary medicine in this collection. However, the paper by Arvidsson et al. (22) focuses on the interface between bio-veterinary medicine and vernacular veterinary knowledge, using the notion of paraprofessionals to describe those whose knowledge about animals is recognized not only by the local animal owners but also by veterinarians in the field, even though it doesn't take the form of academic science. In the context of privatization of veterinary services and limited access to qualified veterinary care, one can become a paraprofessional veterinarian by acquiring certificates or diplomas in, for example, general agriculture or animal management, or by being trained for a few months by an NGO. The inclusion of paraprofessionals concerns the very structure of the veterinary profession, which was founded on its distinction from the empirical knowledge carried and transmitted by the many lay animal experts working as farmers, butchers, blacksmiths, etc. (23). Arvidsson et al. (22) show that this tension increases when small farmers turn to paraprofessionals to meet their small budgets, while veterinarians encourage them to increase the size of their farms. Arvidsson et al. (22) also insist that, because pig farming is recent in Uganda, smallholders don't have traditional knowledge on animal diseases, but need a quick and cheap diagnosis on how to cure a disease that ravages their farm. The notion of paraprofessionals seems to have purchase beyond the case described by Arvidsson et al. (22), while the dynamics and relations with university trained veterinarians can be different elsewhere. In China, for example, “duck doctors” are consulted by farmers to provide antibiotics and antivirals against poultry diseases, yet they are suspected to be cheaters or quacks by official veterinarians who practice surveillance for avian influenza and report cases to the authorities (24). These “duck doctors” are closer than official veterinarians to the different scales of poultry farming, from big industrial farms to small poultry farms. Small poultry breeders, often mixing wild and domestic poultry, contend that small scale farming produces a form of immunity toward avian influenza, which, in their view, only affects big industrial farms.

While risk culture and ethno-science are two productive modes of collaboration between veterinarians and anthropologists, their respective scientific knowledge-making activity remains separated. A more recent turn in anthropology has led anthropologists to describe how veterinarians can work with animals, farmers, and retailers to increase the health of the community, which leads, in our view, to a more fruitful form of collaboration between life sciences and social sciences.

## 3. Animals in infrastructures

Veterinarians address questions on animals through the frame of their medical practice, and anthropologists can elaborate these questions in the temporal and spatial variations of human-animal relations. Veterinarians are good partners for collaboration with anthropologists because they are mediators between a plurality of views on animals. In collaboration with anthropologists, these views become synthesized into an encompassing knowledge. When interacting with veterinarians as subjects of fieldwork, anthropologists are reminded that animals are also subjects who can disrupt protocols of care and cure. There are thus two tendencies in contemporary anthropology that can enter into tension with each other around the care of animals: looking at infrastructures and displaying agencies.

### 3.1. Agency and attachment

Anthropology has long viewed animals as carriers of symbols and meanings, and humans as the only agents of cultural production. Animals and plants were considered the background environment on which humans elaborated their worldviews. In the last decades, several initiatives in anthropology have led to viewing animals as agents interacting with humans in fundamental dimensions of social life. This tendency, called “the animal turn in anthropology,” echoes the sensitivity to the concept of animality in philosophy and many other fields of academic production.

Anthropology has questioned how the notion of nature has emerged to gather animals and plants under an objective gaze (25). It points to the variety of forms of human-animal relations that do not pass through this framework. For instance, in Amazonia, hunters must adapt the perspective of the animals they prey on, treat them as kin, and ask for their consent. For Amazonian shamans, “to know is to personify, to take the point of view of that which must be known” (26). The ontological turn consists in taking these discourses seriously, not as metaphors projecting human relations on animal behaviors, but as statements in which humans “are” or “become” animals. Indeed, Amazonian myths tell stories of a time when humans and animals were unified and explain their separation as a family conflict. This raises the difficult question of whether animals can be considered agents if they don't have language. Kohn (27) has argued that language has obfuscated the question of animal agency, because it remains at the level of conventional symbols. But the hunting practices he observed in Ecuador connected humans and animals instead through signs: for instance, it is possible to anticipate the future of human-animal encounters by being attentive to the sounds of trees. A biosemiotic of animals looks at how they communicate across species by sharing signs beyond conventional symbols. Candea (28) has transposed these Amazonian observations into the world of evolutionists, who must qualify animal behaviors using human terms such as “agree” or “exchange.” These Western scientists have one theory of behavior which they divide among several species, just as Amazonian hunters have one theory of the soul which is diffracted among different species. For Amazonians as for behaviorists, humans and animals have the same soul but differ by their respective bodies. The problem for behaviorists is how to shift from moments when humans and animals share behaviors to moments when they need to be different species: for instance, euthanasia of experimental animals is described as passing from attachment to detachment (29, 30). When veterinarians face ethical dilemmas in the management of animal death, they use forms of thinking that are common in non-Western societies and that have been marginalized by biomedical science (6).

Anthropology is thus moving away from the central figure of the human to open its description to the multiplicity of living beings with whom humans interact. In the words of Haraway (31), anthropologists are now led to consider animals not only as good to eat or good to think but also as good to live with. Haraway (31) has coined the term “companion species” to point to kinship relations by which humans and animals exchange substances and affects. Following her indications, at the crossroads of science studies and animal studies, a collective of anthropologists (32) has developed the method of multi-species ethnography, which doesn't limit itself to charismatic animals such as primates but also considers small beings such as plants, microbes, and fungi. Symbiotic relations between humans, animals, and microbes are privileged over warning signals on zoonotic pathogens because they make up the “viral chatter” or “viral clouds” out of which these emergences take shape.

Ashall (33) quotes this literature to think about her own practice as a veterinarian engaged in a difficult clinical situation that involved euthanizing a dog. This situation entailed not only therapeutic decisions but also challenges for the communicative and emotional interactions with the dog and their owners. Ashall (33) uses Haraway (31) concept of tentacularity to understand how animals connect humans to other living beings in ways that make them responsible, that is, able to respond to questions about life and death. Proposing the concept of “emotional sponge” and relying on a feminist ethics of care, she shows that these reactions are different from the responses that are expected from veterinarians in their training. While the animal turn in anthropology may appear as too philosophical or literary, it can be connected to the problems that veterinarians meet in their practices when they consider animals as agents and not only as patients—that is, when they take seriously their capacity to interpellate those who take care of them.

### 3.2. Infrastructures and biosecurity

Another way to bring veterinarians into anthropological description is to see how they are inscribed in infrastructures of knowledge such as farms, natural reserves, laboratories, or clinics. By infrastructures, we mean material configurations enabling regimes of care and security. When veterinarians take care of animals, when they diagnose a zoonosis, they are part of an infrastructure that organizes forms of care but that can also be transformed by the anticipation of the future. When anthropologists follow veterinarians, they can ascribe agency to animals by a method of multispecies ethnography, but they also observe the systematic constraints of the infrastructures, in what has been called second-order observation (34). Infrastructures change the way animal lives, diseases, and deaths are visualized and problematized. The difference between professional veterinarians and paraprofessionals, to take the case described by Arvidsson et al. (22), is not only a difference in knowledge and way of life, but also a difference in access to infrastructures such as clinics and labs, which veterinarians use as practitioners but also constitute with other institutional actors.

When veterinarians ask how farmers or retailers perceive the risk of zoonoses, they focus on mental and social conditions of life without considering the infrastructures in which humans interact with animals. Anthropology, together with sociology and social geography, has described how biosecurity has changed the conditions of work with animals when it was transferred from laboratories to markets and farms under the framework of emerging infectious diseases (35, 36). Biosecurity is a technique of risk management or anticipation of the future that does not rely on the calculation of the probability of industrial accidents, but rather imagines the effects of catastrophic events. Under the rules of biosecurity, farmers and retailers are required to imagine that a new virus or bacterium has entered their working space and could escape to other spaces where it could cause disastrous outbreaks—hence the need to build barriers against spaces where animals are raised but also to prepare for what could happen should these barriers be crossed. In poultry farms in Hong Kong, some chickens are not vaccinated so they may raise the alert in the presence of the influenza virus, serving as sentinels—the Chinese term literally means “chickens whistling like soldiers” (37). On pig farms in the Midwest, workers must follow biosecurity measures to avoid transmitting influenza to pigs (38). In both cases, the management of zoonoses under rules of biosecurity produces new forms of exclusion, but also solidarity between humans and animals.

Thinking about veterinary anthropology in terms of infrastructures for human-animal relations is important for understanding how biomedicine has extended beyond local sites where humans interact with animals. Science studies have shown how the laboratory has played a major role in connecting farms and clinics, veterinarians and physicians, through the visualization of the microbe (39, 40). Veterinary knowledge has been used to produce vaccines from cows or chickens and serums from horses, making connections with the livestock industry and contributing to the pharmaceutical industry. The capacity to produce the same serum or vaccine in laboratories displaced across heterogeneous spaces, from city to countryside, from metropole to colonies, has contributed to the success of microbiological knowledge. These traveling forms of measurement find a new significance with contemporary techniques of “One Health” that connect human and animal health in global databases. The surveillance of the emergence of zoonotic pathogens allows veterinarians across the globe to communicate through a shared language of biosecurity, but might lead to a separation between veterinarians and the local practices that make health possible (41).

Hence the need for a critical reflection on how veterinary knowledge has been extended across species and territory borders to understand how it can meet with anthropological knowledge. Anthropologists made a reflexive turn when they discovered that the subjects they encountered were actors in a global system of power, in which anthropological knowledge could be used (42, 43). In the same way, focusing on animals as agents in biosecurity infrastructures should lead veterinary knowledge to understand how it already has become global, rather than requesting its globalization under the motto “One Health.” If anthropologists are experts in social participation (how living beings assemble together), they are also experts in reflexivity (how to think about the separation between self and other). Veterinary anthropology, beyond calls for the participation of stakeholders, should also be a reflection on how animal sciences became global as part of what is now called global health.

## 4. Anthropology of veterinarians

In exploring and imagining the emerging field of veterinary anthropology, we proceed in the final section to direct the critical, analytical faculty of anthropology toward veterinary expertise itself as a practice in more-than-human worlds. Put differently, we want to further the proposal that “anthropology with veterinarians” benefits from being ultimately also an “anthropology of veterinarians.”

### 4.1. The growing role of veterinarians in contemporary societies

To set out the argument, we return to what medical anthropology taught us about the role of human medicine in contemporary societies. It has been convincingly argued by scholars in social sciences and humanities, as well as public intellectuals at large, that over the last century or two, many “behaviors that were once defined as immoral, sinful, or criminal have been given medical meaning, moving them from badness to sickness” (44). Simultaneously, the argument goes, many common processes of human life have also been recast as medical conditions. Hence “anxiety and mood, menstruation, birth control, infertility, childbirth, menopause, aging, and death” (44) are now diagnosed, monitored, and treated. This process, in which medicine gains seemingly ever-growing influence over our lives, has been coined “medicalization of society.” Labeling the process, of course, does not in itself explain much [see (45)], yet it directs reflexive social scientific attention. In other words, naming the process generated the need and opportunity to study it, which led to a robust reflexive discourse on medicalization [to mention just a few, (44–51)]. We suggest that bio-veterinary medicine, one that we can also term technoscientific, exercises increasing and global influence over human and animal lives, and we propose to call this process “veterinarization of society” (52). At first glance, this might seem a farfetched notional parallel to “medicalization of society,” yet, upon closer inspection, this process seems hard to overestimate.

We saw in the Arvidsson et al. (22). contribution to this collection how veterinary researchers and field veterinarians in Uganda promote the business-minded reorientation of small-holder production to tackle rural poverty. Høg et al. (13), in their article from our collection, exemplify a connected ambition of veterinary professionals to foster not only efficiency and “plenty” but “safety” in handling animals and their bodies along the food chain. They convincingly argue that, in order to decrease the risk of pathogen emergence and transmission, structural features of Bangladeshi live animal commodity chains must be addressed. In many other places, veterinary concerns are already well integrated into socio-material infrastructures and established practices and policies. Staying with the Høg et al. (13) example of avian influenza, we can point to what Keck (37) called techniques of preparedness. Within this framework, relations between humans and birds in places like Singapore or Hong Kong are veterinarized, that is, transformed with the aid of vaccination [see also (53) in this Research Topic], monitoring, mass culling, or biosecuritization of borders.

Food chains that veterinarians foster and regulate are not limited to farm production. The already- mentioned bush meat or wet markets are linked to the emergence of COVID-19 and form a new frontier on which veterinary powers are currently negotiated (54). In Europe, hunting practices are also emerging as the new arena of veterinary surveillance and intervention, among other reasons because of the threat of African Swine Fever (52, 55). Here, too, borders are bio-securitized with the aid of veterinary infrastructures such as actual or planned fences, veterinary surveillance becomes part of various trades' canons, etc. Another subject of increasing veterinary intervention are companion species, due to their actual and potential role in pathogen spread to humans and/or livestock. An example of a concern on that front is discussed by Hobson-West's (53) contribution that focuses on human owners' resistance to their pets' vaccinations and connected worries of veterinary specialists.

Veterinary logic extends still further, beyond concerns about efficiency and safety. Animal welfare is one of the issues that define the veterinary profession, be it in actual daily veterinary care, or the bureaucratic role of controlling, licensing, etc., or expert witnessing. These roles become more complex when concerns about animal wellbeing stretch across various socio-cultural contexts with their specific takes on animal welfare. Chao's paper (56) in our collection shows veterinary scientists called upon by policymakers and the meat industry to make the “Western” humane slaughter requirement of stunning compatible with the halal slaughter that allows “no harm” prior to the throat being cut. In this context, veterinarians are becoming cultural brokers, involved in non-trivial mediation between distinct frameworks of animal ethics.

Already these few examples derived from the present special issue illustrate well that, in many contexts, veterinary experts inform, format, and structure modes of human-animal relations in fundamental ways. Whether we look at caring for, killing, or consuming of animals, veterinary medicine not only offers knowledge and assistance but, teamed up with the law-making and executive branches of the state, often also demonstrates an ambition to prescribe, regulate, and sanction for the sake of animal and human wellbeing alike. Put bluntly, in many parts of the world veterinarians are powerful experts with a growing jurisdiction that extends across various arenas of bio-politics. This movement goes far beyond the tension we have described between local knowledges and biomedicine, raising new forms of animal agency in infrastructures of knowledge.

### 4.2. The sociology of veterinarians

However, the process evoked above that we coined “veterinarization of society” stands in striking contrast to the self-perception of veterinary specialists as described by Desmond [(57) in this Research Topic]. She demonstrates how American veterinarians feel marginalized, undervalued, and powerless, in constant comparison with human medicine doctors. How can we explain this contradiction of veterinarians gradually gaining powers while feeling marginalized? And how widespread is this professional self-image globally?

Giving a comprehensive answer is hard. Since 2013, CM Research Ltd. has been conducting an annual global survey of veterinarians (58), and in 2020 it teamed up with WSAVA (World Small Animal Veterinary Association) and several local veterinary associations for this goal. The survey covers a number of issues, from the demography of veterinary specialists (including gender and age) to the size of the practice and the ratio of veterinarians to veterinary nurses and technicians. It also addresses more nuanced questions such as respondents' outlook on the future or level of job satisfaction. This survey is admittedly biased in favor of companion animal veterinarians who form the majority of respondents, while only a “small proportion of large animal veterinarians has also taken part” in the survey (58). Even more importantly, despite being global in their claims, of the 5,000 veterinary professionals from 91 countries who participated in one of the last surveys to reveal the impact of the COVID-19 pandemic on the veterinary market, only 32 came from Africa and 132 from Asia (59).

Academic literature seems to give a richer, more nuanced picture, well visible in a recent review of social scientific research on the veterinary profession given by Bonnaud and Fortané (60). They distinguish four main thematic clusters, after a close reading of more than a hundred works, some of which shed light on trends pinpointed also in the above-mentioned surveys. First is the sociodemographic evolution of the profession, which is undergoing “dramatic, rapid feminization,” while “in many ways remaining gendered masculine” in actions and attitudes (61). Second is the massive growth of small animal healthcare leading to a situation in which “the majority of veterinarians specialize in pet care” (60). Third is the ongoing transformation of the agricultural sector, leading to the contradictory trends of a decline in farm animal medicine on the one hand, and a growing significance of veterinarians in industrial (poultry and pig) farming on the other hand, in the dual capacity of “health managers” fostering the “technico-economic objectives of productivity and profitability” and dealers in the veterinary drug market (60). Finally, the fourth theme is the changing role of veterinarians in the public health state, reformed as part of the neoliberal turn (62), which might, at least partly, explain the paradox of the empowerment of veterinarization processes paired with the subjectively perceived powerlessness of veterinary specialists.

However, as Bonnaud and Fortané admit, their “overview is geographically limited: the research focuses essentially on west-European countries and the USA” (60). The authors are careful, wondering whether it is not the choice of languages (English and French) that determines the areas covered in the reviewed literature. Adding German, Czech, Slovak, and Russian, we tend to believe that the problem is not the selectivity of the review's authors, but of the total body of existing scholarship. Most social scientific work on the veterinary profession deals with Euro-America and sometimes even implicitly generalizes in a way that unconsciously treats the bio-veterinary medicine of the global North as a default form [for a similar claim about such bias in knowledge production, see for example (63)]. Thus, despite some valuable studies addressing veterinary realities beyond Euro-America (23), the comparative sociology of veterinary professions seems rather limited, and so is our idea of the role of veterinary experts and expertise on a global scale, let alone about its ongoing changes and paradoxes.

Of course, the factor to be taken into account is how widespread the veterinary profession is; could not the reason for knowing comparatively much less about veterinary professionals beyond Euro-America be that there is simply much less veterinary medicine going on there? The proxy data we were able to find show that even in Europe there is rather significant variation in the number of veterinarians. The number of active veterinarians per 1,000 of the human population ranges from 1.29 in Latvia to 0.13 in Northern Macedonia (the European average is 0.35) (64). This is a rough indicator, as it does not take into account how many animals these veterinarians care for, what tasks they cover, or what veterinary infrastructures they have at hand; yet, for most of the world, we do not have even this crude indicator available. Still, there are indications that most countries have less, or even much less, active veterinarians than the European average (19). We believe, however, that this does not mean that animal health is not an issue people constantly care about. Rather, it finally leads us to one of the crucial questions of the emerging veterinary anthropology, in contrast to a sociology of veterinarians, namely: Who is a veterinary expert in transcultural perspective?^3^

### 4.3. Challenges for veterinarians in global health

By veterinary medicine, we often understand the Western system of knowledge and set of practices, created, maintained, and developed by veterinary experts and entrenched in infrastructures (institutional, legal, etc.), that became global largely as a result of (post)colonial expansions. The role and value of veterinary anthropology as we understand it should nevertheless consist in the critical ability to “provincialize” this seemingly universal expert system, showing that it is one of many by exposing its historicity, limits, and also past and present competitors. On that front of “provincializing,” we see three main areas to be systematically explored:

First, in dialogue with the related fields of ethno-veterinary medicine and classical anthropology of animal agency (6), veterinary anthropology should focus on the whole repertoire of vernacular practitioners and practices related to animal health and illness. These can vary from isolated pockets of local knowledge and conduct exercised by nearly any member of a given community to elaborate veterinary cultures of valued specialists that are (as with Greco-Roman antiquity) or are not [as with the medieval Arabic sources; cf. (65)] seen as part of the genealogy of Western veterinary medicine. The task of veterinary anthropology is a symmetrical treatment of ideas, practices, and practitioners dealing with animal health and illness, regardless of where they are standing vis-à-vis Western bio-veterinary medicine in its current form.

Second, veterinary anthropology is in a good position to pay detailed attention to the actual processes of globalization of Western bio-veterinary medicine. What are the incentives and networks of power relations, as well as the consequences for the socio-ecologies of human-animal relations that are recently falling under the jurisdiction of bio-veterinary medicine? Sometimes these can be radical, leading to “villainization” of some species in local contexts, such as poultry raised on the rooftops of Cairo being killed because of the risk of avian influenza (36). A connected question, then, is what globalization does to bio-veterinary medicine itself. It seems beyond doubt that successful diffusion always comes at the cost of change and diversification of the entity that successfully travels. Anthropologists have described that for Christianity (66–69) and bio-medicine (70), while already existing studies, including those collected in this special issue, bear witness to the cultural localizations of bio-veterinary medicine in various contexts. The search for veterinary definitions of halal slaughter is just one, albeit catchy, example of what could be the tasks of bio-veterinary medicine moving beyond Euro-American contexts (56). Thus, upon closer examination, the seemingly monolithic rationality of bio-veterinary medicine is likely to reveal itself as a colorful tapestry, because it is always “a product of specific institutional contexts that might look different in other contexts, places and times” (62).

Third, veterinary anthropology needs to cultivate sensitivity to dissenting knowledges and practices that thrive in the so-called West. Here again, medical anthropology teaches us a useful lesson. Just as complementary and alternative medicine (CAM) is a well-established topic of medical anthropology, seen as an indispensable part of the jigsaw puzzle of understanding the lived world of human health and illness, so should complementary and alternative veterinary medicine (CAVM) be understood as an indispensable part of our understanding of human-animal relations unfolding around animal health and illness. As Klepal and Stöckelová argue, CAM practices are not simply either in opposition to biomedicine or tamed and incorporated into it. In their case-study of Chinese medicine adoption in the contemporary Czech Republic, they demonstrate that “the CAM practices … can also play a pioneering role in advancing some of the processes [often] described [by researchers] as ‘biomedicalization”' (50). Namely, they talk about reshaping patient subjectivities or promoting the concept of a person's “inborn individual constitution” (50). In this vein, we can expect that thorough empirical engagement would uncover more nuanced relations between CAVM and bio-veterinary medicine, including “bio-veterinarization beyond bio-veterinary medicine” [to paraphrase (50)]. Furthermore, we can anticipate multidirectional flows of ideas, practices, and attitudes between CAVM and various vernacular approaches to animal health and illness that form yet another dimension of their relation with bio-veterinary medicine and processes of veterinarization.

## 5. Conclusion

We can conclude that the processes of the veterinarization of society, that is, extensions of veterinary “jurisdiction, authority, and practice into increasingly broader areas of people's lives” (71), reshape relations between humans and animals far beyond the opposition between global bio-medicine and local knowledges (50). The role of various specialists of animal health, from local animal healers to academic veterinarians, in these processes is far from clear, awaiting thorough empirical engagement. Only then we can attempt to decipher the paradox of veterinarians feeling marginal amid the accelerating global processes of veterinarization. This special issue thus calls for empirical and reflexive accounts of sites where veterinarians interact with potentially sick animals and other types of animal caregivers to describe the ethical and political challenges they meet when they are in charge of more-than-human health.

This vision of veterinary anthropology, anchored in the broader field of veterinary humanities, draws on the experience of medical/health studies and their relation to human medicine. The concept of “veterinarization of society” that we introduced in the previous section should not be understood as an attack on veterinary expertise. Quite to the contrary, recognizing veterinary expertise in a variety of its forms as both powerful and important requires critical reflection on its role in contemporary societies, something we have coined “anthropology of veterinarians.” Such critical reflection is then a valuable starting point for further developing veterinary anthropology as “anthropology with veterinarians,” a joint, truly collaborative enterprise that contributes to better understanding of, and participation in, the world that has always been more-than-human.

## Author contributions

All authors listed have made a substantial, direct, and intellectual contribution to the work and approved it for publication.

## Footnotes

^1^See https://veterinary-humanities.blogspot.com/2020/08/doing-animal-health-in-more-than-human.html.

^2^ http://www.medanthrotheory.org/article/view/5659/7476

^3^This question of course should be juxtaposed to asking who is a potential patient of such expert, i.e., which animals qualify for that attention—see Desmond, this special issue x research topic.

## References

[1] Brown H Nading AM. Introduction: human animal health in medical anthropology. Med Anthropol Q. (2019) 33:5–23. 10.1111/maq.12488 30811674 PMC6492111

[2] Asdal K Druglitro T Hinchliffe S. Humans, Animals and Biopolitics: The More-Than-Human Condition. London: Routledge (2016). p. 195. 10.4324/9781315587639

[3] Donald MM. When care is defined by science: Exploring veterinary medicine through a more-than-human geography of empathy. Area. (2019) 51:470–8. 10.1111/area.12485

[4] Weich K Grimm H. Meeting the patient's interest in veterinary clinics. Ethical dimensions of the 21st Century animal patient. Food Ethics. (2018) 1:259–72. 10.1007/s41055-017-0018-0

[5] Ingold T. Editorial. Man (NS). (1992) 27:693–6.

[6] Keck F. A genealogy of animal diseases and social anthropology (1870–2000). Med Anthropol Q. (2019) 33:24–41. 10.1111/maq.12442 29572952

[7] Keck F. Veterinary anthropology: When medical anthropology meets animal studies MAT. (2016). Available online at: http://www.medanthrotheory.org/article/view/5659/7476 (accessed November 6, 2018).

[8] Banwell C Ulijaszek S Dixon J. When Culture Impacts Health: Global Lessons for Effective Health Research. 1st edition. London, Waltham, MA: Academic Press. (2013). p. 380. 10.1016/B978-0-12-415921-1.00001-4

[9] Parveen S Islam MS Begum M Alam MU Sazzad H Sultana R. It's not only what you say, it's also how you say it: communicating nipah virus prevention messages during an outbreak in Bangladesh. BMC Public Health. (2016) 16:1–11. 10.1186/s12889-016-3416-z 27495927 PMC4974711

[10] Hegde ST Sazzad H Hossain MJ Alam MU Kenah E Daszak P. Investigating rare risk factors for Nipah virus in Bangladesh: 2001–2012. Ecohealth. (2016) 13:720–8. 10.1007/s10393-016-1166-0 27738775 PMC5164848

[11] Brown H Kelly AH. Material proximities and hotspots: toward an anthropology of viral hemorrhagic fevers. Med Anthropol Q. (2014) 28:280–303. 10.1111/maq.12092 24752909 PMC4305216

[12] Douglas M Wildavsky A. Risk and Culture: An Essay on the Selection of Technological and Environmental Dangers. Berkeley, California: University of California Press. (1983). p. 224. 10.1525/9780520907393

[13] Høg E Fournié G Hoque MdA Mahmud R Pfeiffer DU Barnett T. Avian influenza risk environment: live bird commodity chains in Chattogram, Bangladesh. Front Veter Sci. (2021) 8:694753. 10.3389/fvets.2021.694753 34616791 PMC8489835

[14] Keck F Lynteris C. Zoonosis: prospects and challenges for medical anthropology. Med Anthropol Theory. (2018) 5:1–14. 10.17157/mat.5.3.372

[15] McCorkle CM Mathias-Mundy E. Ethnoveterinary medicine in Africa. Africa. (1992) 62:59–93. 10.2307/1160064

[16] Eiki N Maake M Lebelo S Sakong B Sebola N Mabelebele M. Survey of ethnoveterinary medicines used to treat livestock diseases in omusati and kunene regions of Namibia. Front Veter Sci. (2022) 9:762771. 10.3389/fvets.2022.762771 35296062 PMC8920552

[17] Balick MJ Cox PA. Plants, People, and Culture: The Science of Ethnobotany. 2nd ed. New York: Garland Science. (2020). p. 228. 10.4324/9781003049074

[18] Khan K Rahman IU Calixto ES Ali N Ijaz F. Ethnoveterinary therapeutic practices and conservation status of the medicinal flora of Chamla valley, Khyber Pakhtunkhwa, Pakistan. Front Veter Sci. (2019) 6:122. 10.3389/fvets.2019.00122 31157243 PMC6532436

[19] Tiwari L Pande PC. Ethnoveterinary medicines in Indian perspective: reference to Uttarakhand, Himalaya. IJTK. (2010) 9:611–7.

[20] Jacob MO Farah KO Ekaya WN. Indigenous knowledge: the basis of the Maasai Ethnoveterinary diagnostic skills. J. Human Ecol. (2004) 16:43–8. 10.1080/09709274.2004.11905714

[21] Lainé N. Living and Working With Giants: A Multispecies Ethnography of the Khamti and Elephants in Northeast. Paris: MNHN. (2020). p. 272. 10.5852/nes02

[22] Arvidsson A Fischer K Hansen K Sternberg-Lewerin S Chenais E. Diverging discourses: animal health challenges and veterinary care in northern Uganda. Front Veter Sci. (2022) 9:773903. 10.3389/fvets.2022.773903 35359673 PMC8960384

[23] Hubscher RH. Les maîtres des bêtes: les vétérinaires dans la société française, XVIIIe-XXe siècle. Odile Jacob; (1999). p. 452.

[24] Fearnley L. Virulent Zones: Animal Disease and Global Health at China's Pandemic Epicenter. S.l.: Duke University Press Books; (2020). p. 296. 10.1215/9781478012580

[25] Descola P. Beyond Nature and Culture. Chicago, London: University of Chicago Press. (2013). 10.7208/chicago/9780226145006.001.0001

[26] de Castro EBV. Exchanging perspectives: the transformation of objects into subjects in Amerindian ontologies. Common Knowl. (2004) 10:463–84. 10.1215/0961754X-10-3-463

[27] Kohn E. How Forests Think Toward an Anthropology Beyond the Human. Berkeley: University of California Press. (2013). 10.1525/9780520956865

[28] Candea M. Different species, one theory: reflections on anthropomorphism and anthropological comparison. Cambridge Anthropol. (2012) 30:118–35. 10.3167/ca.2012.300208

[29] Candea M. “I fell in love with Carlos the meerkat”: Engagement and detachment in human–animal relations. Am Ethnol. (2010) 37:241–58. 10.1111/j.1548-1425.2010.01253.x

[30] Sharp LA. Animal Ethos: The Morality of Human-Animal Encounters in Experimental Lab Science. California: University of California Press. (2018). p. 310. 10.1525/california/9780520299245.001.0001

[31] Haraway D. When Species Meet. Minneapolis: University of Minnesota Press. (2008).

[32] Kirksey SE Helmreich S. The emergence of multispecies ethnography. Cultur Anthropol. (2010) 25:545–76. 10.1111/j.1548-1360.2010.01069.x

[33] Ashall V. A feminist ethic of care for the veterinary profession. Front. Veter. Sci. (2022) 9:795628. 10.3389/fvets.2022.795628 35372559 PMC8970040

[34] Gershon I. Seeing like a system: Luhmann for anthropologists. Anthropol Theory. (2005) 5:99–116. 10.1177/1463499605053993

[35] Lakoff A Collier SJ. Biosecurity Interventions: Global Health and Security in Question. New York, NY: Columbia University Press. (2008). 10.7312/lako14606

[36] Hinchliffe S Bingham N. Securing life: the emerging practices of biosecurity. Environ Plann A. (2008) 40:1534–51. 10.1068/a4054

[37] Keck F. Avian Reservoirs: Virus Hunters and Birdwatchers in Chinese Sentinels Posts. Durham: Duke University Press. (2020). 10.1215/9781478007555

[38] Blanchette A. Porkopolis: American Animality, Standardized Life, and the Factory Farm. Durham: Duke University Press. (2020). 10.1215/9781478012047

[39] Latour B. The Pasteurization of France. Cambridge, London: Harvard University Press. (1988).

[40] Robinson PA. Performativity and a microbe: Exploring Mycobacterium bovis and the political ecologies of bovine tuberculosis. Biosocieties. (2019) 14:179–204. 10.1057/s41292-018-0124-1 32226469 PMC7100403

[41] Hinchliffe S. More than one world, more than one health: Re-configuring interspecies health. Soc Sci Med. (2015) 129:28–35. 10.1016/j.socscimed.2014.07.007 25022470

[42] Robinson PA. Framing bovine tuberculosis: a ‘political ecology of health' approach to circulation of knowledge(s) about animal disease control. Geogr J. (2017) 183:285–294. 10.1111/geoj.12217

[43] Hausermann HE. ‘I could not be idle any longer': buruli ulcer treatment assemblages in rural Ghana. Environ Plann A. (2015) 47:2204–20. 10.1177/0308518X15599289

[44] Conrad P. The Medicalization of Society: On the Transformation of Human Conditions into Treatable Disorders. Baltimore: Johns Hopkins University Press. (2007). p. 224.

[45] Rose N. Beyond medicalisation. Lancet. (2007) 369:700–2. 10.1016/S0140-6736(07)60319-5 17321317

[46] Zola IK. Medicine as an institution of social control. Sociol Rev. (1972) 20:487–504. 10.1111/j.1467-954X.1972.tb00220.x 4645802

[47] Parens E. On good and bad forms of medicalization. Bioethics. (2013) 27:28–35. 10.1111/j.1467-8519.2011.01885.x 21535062

[48] Illich I. The medicalization of life. J Med Ethics. (1975) 1:73–7. 10.1136/jme.1.2.73 809583 PMC1154458

[49] Seeberg J. The Event of DOTS and the Transformation of the Tuberculosis Syndemic in India. The Cambridge J Anthropol. (2014) 32:95–113. 10.3167/ca.2014.320108

[50] Klepal J Stöckelová T. Exploring biomedicalization through complementary and alternative medicine in a postsocialist context. Med Anthropol. (2018) 37:412–25. 10.1080/01459740.2018.1473395 29924640

[51] Clarke AE Mamo L Fosket JR Fishman JR Shim JK. Biomedicalization: Technoscience, Health, and Illness in the U.S. Illustrated edition. Durham, NC: Duke University Press Books. (2010). p. 512. 10.2307/j.ctv125jk5c

[52] Broz L Arregui AG O'Mahony K. Wild boar events and the veterinarization of multispecies coexistence. Front Conser Sci. (2021) 2:110. 10.3389/fcosc.2021.711299

[53] Hobson-West P. Vets and Vaccines: A discursive analysis of pet vaccine critique. Front. Veter. Sci. (2022) 9:868933. 10.3389/fvets.2022.868933 35754533 PMC9218416

[54] Lynteris C. The Imperative Origins of COVID-19. L'Homme. (2020) 234:21–32. 10.4000/lhomme.37062

[55] Emond P Bréda C Denayer D. Doing the “dirty work”: how hunters were enlisted in sanitary rituals and wild boars destruction to fight Belgium's ASF (African Swine Fever) outbreak. anth. (2021) 56:87–104. 10.5252/anthropozoologica2021v56a6

[56] Chao EC. Islam and Veterinary Science: rethinking animal suffering through islamic animal ethics and the evolving definition of halal slaughter. Front. Veter. Sci. (2022) 9:785585. 10.3389/fvets.2022.785585 35664849 PMC9159294

[57] Desmond J. Medicine, value, and knowledge in the veterinary clinic: questions for and from medical anthropology and the medical humanities. Front. Veter. Sci. (2022) 9:780482. 10.3389/fvets.2022.780482 35359687 PMC8964002

[58] Moorcock A Potter N Kunduz-Kara D. VetsSurvey 2021: understanding the veterinary profession Vetspanel - owned managed by CM Research. (2022). Available online at: https://www.portalveterinaria.com/upload/20220127112329Vet-Survey-2021-Final-compressed.pdf (accessed March 29, 2022).

[59] VetsSurvey 2020. Part 1 - COVID-19 Global Pandemic impact on the veterinary market Vetspanel - owned managed by CM Research. (2022). Available online at: https://www.cm-research.com/covid-19-global-pandemic-impact-on-the-veterinary-market/ (accessed March 29, 2022).

[60] Bonnaud L Fortané N. Being a vet: the veterinary profession in social science research. Rev Agric Food Environ Stud. (2021) 102:125–49. 10.1007/s41130-020-00103-1

[61] Irvine L Vermilya JR. Gender work in a feminized profession: The case of veterinary medicine. Gender Soc. (2010) 24:56–82. 10.1177/0891243209355978

[62] Enticott G Lowe P Wilkinson K. Neoliberal reform and the veterinary profession. Vet Rec. (2011) 169:327. 10.1136/vr.d5384 21949202

[63] Jehlička P. Eastern Europe and the geography of knowledge production: The case of the invisible gardener. Progress Human Geogr. (2021) 45:1218–36. 10.1177/0309132520987305

[64] Survey of the veterinary profession in Europe Federation of Veterinarians of Europe. (2019). Available online at: https://fve.org/cms/wp-content/uploads/FVE_Survey_2018_WEB.pdf (accessed March 29, 2022).

[65] Shehada HA. Mamluks and Animals: Veterinary Medicine in Medieval Islam. Illustrated edition. Boston: BRILL. (2012). p. 592.

[66] Hefner RW. Conversion to Christianity: Historical and Anthropological Perspectives on a Great Transformation. California: University of California Press. (1993). p. 344. 10.1525/9780520912564

[67] Engelke M. God's Agents: Biblical Publicity in Contemporary England. California: University of California Press; (2013). p. 320 p. 10.1525/california/9780520280465.001.0001

[68] Chua L. The Christianity of Culture: Conversion, Ethnic Citizenship, and the Matter of Religion in Malaysian Borneo. Palgrave Macmillan; (2012). p. 275 p.

[69] Robbins J. Becoming Sinners. Christianity + Moral Torment in a Papua New Guinea Society. Berkeley, Los Angeles, London: University of California Press. (2004).

[70] Lock MM Nguyen VK. An Anthropology of Biomedicine. New York: John Wiley & Sons. (2018). p. 555.

[71] Clarke AE Shim JK Mamo L Fosket JR Fishman JR. Biomedicalization A TheoreTiCAl And SUbSTAnTive inTrodUCTion. In: Clarke AE Mamo L Fosket JR Fishman JR Shim JK editors. Biomedicalization: Technoscience, Health, and Illness in the US. Illustrated edition. Durham, NC: Duke University Press Books (2010). p. 1–44. 10.2307/j.ctv125jk5c.5

